# Prevalence and predictors of non-alcoholic liver disease on MRI among patients with Crohn's disease

**DOI:** 10.1186/s12876-022-02238-5

**Published:** 2022-04-12

**Authors:** Qijin Hong, Jun Shen, Qi Feng, Qing Zheng, Yuqi Qiao

**Affiliations:** 1 Division of Gastroenterology and Hepatology; Shanghai Institute of Digestive Disease; NHC Key Laboratory of Digestive Diseases; Inflammatory Bowel Disease Center, Renji Hospital, School of Medicine, Shanghai Jiao Tong University, 160 Pu Jian Road, Shanghai, 200127 China; 2 Department of Radiology, Renji Hospital, School of Medicine, Shanghai Jiao Tong University, 160 Pu Jian Road, Shanghai, 200127 China

**Keywords:** Crohn’s disease, Non-alcoholic fatty liver disease, Magnetic resonance imaging, Inflammatory bowel disease, Prevalence, Prediction

## Abstract

**Background:**

It has been documented that Crohn’s disease (CD) patients were prone to develop non-alcoholic liver fatty liver disease (NAFLD) with less metabolic factors. Our purpose is to investigate the prevalence, clinical characteristics and possible indicators for NAFLD in a cohort of Chinese patients with CD.

**Methods:**

Established CD patients who underwent magnetic resonance enterography (MRE) at the gastroenterology unit of our hospital were consecutively enrolled between June 2018 and May 2020. The diagnosis of NAFLD was made by magnetic resonance proton density fat fraction (MR-PDFF) maps. Medical records during hospitalization were collected and examined by univariate and multivariate analyses. Then a predictive model was constructed based on logistic regression analysis to evaluate the risk of developing NAFLD.

**Results:**

A total of 340 CD subjects were enrolled in this study, 83 (24.4%) suffered from NAFLD. Compared with those without NAFLD, patients with NAFLD showed longer disease duration, higher body mass index (BMI), more frequent use of corticosteroid and pronouncedly elevated liver function tests. The comparison showed no difference in terms of prolonged anti tumor necrosis factor-α (TNF-α) use (> 54w). Multivariate analysis demonstrated that BMI, serum transaminase, pre-albumin and disease duration could independently predict hepatic steatosis.

**Conclusion:**

NAFLD is frequent in chronic CD patients, while long term use of anti TNF-α seems to have no impact on the development of NAFLD in this population. The model incorporating duration, serum transaminase and body mass index presented as a clinical nomogram could well predict the risk of NAFLD in patient with CD.

**Supplementary Information:**

The online version contains supplementary material available at 10.1186/s12876-022-02238-5.

## Introduction

Crohn’s disease (CD) is a subtype of inflammatory bowel disease (IBD) that characterized by discontinuous and ulcerous transmural inflammation of the intestinal tract [1]. Since the “regional ileitis” was formally reported in 1932, there has been over 1, 000,000 people in the United States and 2,500,000 in Europe respectively that estimated to have IBD [2]. Although the etiology of CD remains unclear, the widely use of biological drugs with different mechanisms has change the phenotype of CD [3]. 65–76% of CD patients have been reported to have weight loss, because it can involve any part of the digestive tract, mainly the small bowel [4]. However, increasing observing studies have reported that NAFLD is highly prevalent in CD patients, with less metabolic factors [5, 5]

Non-alcoholic fatty liver disease (NAFLD), a spectrum of liver disorders, affects nearly a quarter of global population, which is also gaining rising incidence in the Asian area [7]. There is now growing evidence that NAFLD is a multi-system disease, which affects both extra-hepatic organs and regulatory pathways [8]. It increases risk of type 2 diabetes mellitus (T2DM), cardiovascular and cardiac diseases, and chronic kidney disease (CKD) [9]. In recent years, mounting data suggest there is an increase in prevalence of NAFLD in IBD population compared with community-based controls [9–12]. McHenry et al. pointed out that Crohn’s disease patients have more than double the risk of NAFLD compared with the general population after considering traditional risk factors [5]. They also constructed a clinical prediction tool to help screen NAFLD, albeit they admitted the limitation of race and ethnicity [15]. Although the pathogenesis of this are complex, some have attributed it to the increasing use of biologics, dysbiosis of microbiota and malnutrition [13]. Notably, Tracey G. Simon et al. identified an association between IRGM gene variation and NAFLD in a large CD cohort [14], which suggests a potentially more complex pathogenesis and relationship between the two diseases.

However, as the discrepancies have been recognized between eastern and western population regarding the phenotypes and natural history of IBD [16], and NAFLD is largely asymptomatic until end-stage complications occur [17], early identification and intervention are pivotal in the management of this co-exist disease. Therefore, the main aim of this article was to evaluate prevalence of NAFLD in CD patients using a Chinese cohort, then to construct a prediction model for risk of NAFLD.

## Design/methods

### Patients

Inpatients diagnosed with CD at the gastroenterology unit of Shanghai Renji Hospital were consecutively enrolled between August 2020 and May 2021. During the hospitalization, each patient underwent magnetic resonance imaging, at the time of which medical records were collected. Clinical parameters including gender, age, disease behavior,BMI, history of medication, as well as laboratory examination results were derived from medical records. The exclusion criteria included excessive alcohol consumption, suspected tuberculosis infection or any other liver diseases. The ethics review board of Renji Hospital, School of Medicine, Shanghai Jiaotong University approved this study (KY-2021-122-B), and informed consent was waived due to the retrospective nature.

### Definition

CD patients in this study were diagnosed based on the ECCO consensus [18] by an multi-disciplinary team (MDT) of experienced experts at our center. Endoscopic and radiological examinations were both taken into account.

The diagnosis of NAFLD was made by using magnetic resonance proton density fat fraction (MR-PDFF), conducted by expert radiologists who has > 5 years of abdominal imaging experience and who were blinded to the clinical history and laboratory details of patients during the time of examination. MR imaging was performed using a 1.5 T GE scanner with an 8-channel, torso phased-array coil (Optima MR360; GE HealthCare, Milwaukee, WI, USA). As for PDFF map, five circular regions of interests (ROIs) about 100 mm^2^ were drawn manually in PDFF maps using the AW4.6 workstation (GE HealthCare, Milwaukee, WI, USA). Among them, three ROIs were evenly placed on right lobe and two on left, avoiding major vessels, ligaments and bile ducts [19]. The mean proportion of liver fat > 5.5% was considered the main outcome of interest [20].

### Construction and validation of a clinical nomogram

We carried out multivariable logistic regression analysis with the following candidate clinical parameters: age, gender, BMI, disease behavior, disease duration, Crohn’s disease activity index (CDAI) and biochemical indicators. We applied backward step-wise selection and used the likelihood ratio test, considering Akaike’s information criterion (AIC) as the ending criteria. AIC is usually performed to assess the goodness of fit of the model. To equip the clinicians with a quantitative tool to predict individual probability of NAFLD, we created a clinical nomogram based on multivariable logistic analysis in this primary cohort.

Discrimination of the prediction models was evaluated by receiver operating characteristics (ROC) analysis and presented as area under the curve (AUC). Odds ratios (OR) having 95% confidence intervals (CI) of final predictors were calculated. Calibration curves were used to assess the calibration of nomograms by comparing the predicted and observed probabilities [21]. The clinical effectiveness of the models was assessed using decision curve analysis (DCA) [22]. The nomogram performance was validated by ten-fold cross-validation with bootstrap.

### Statistical analysis

Data were presented as mean (standard deviations [SDs]) or median (interquartile range [IQRs]) for continuous variables and as number of cases and proportions for categorical ones. We employed multivariable stepwise logistic regression analysis to identify the features that best discriminate NAFLD. The input features consisted of the 9 disease-related parameters and then a feature elimination step was performed with minimum AIC method to select the top features and optimize the model. The operating characteristic curves were plotted and AUC was calculated to assess the diagnostic performance of the model with the pROC package.

Other statistical analysis included non-parametric Mann–Whitney U-test, test of proportions or Pearson chi-square tests. A P value level less than 0.05 was considered nominally significant. All graphic and statistical analyses were conducted either using R statistical software (v4.0.3) or STATA/SE, version 16.

## Results

### Clinical characteristic

**Fig. 1:**
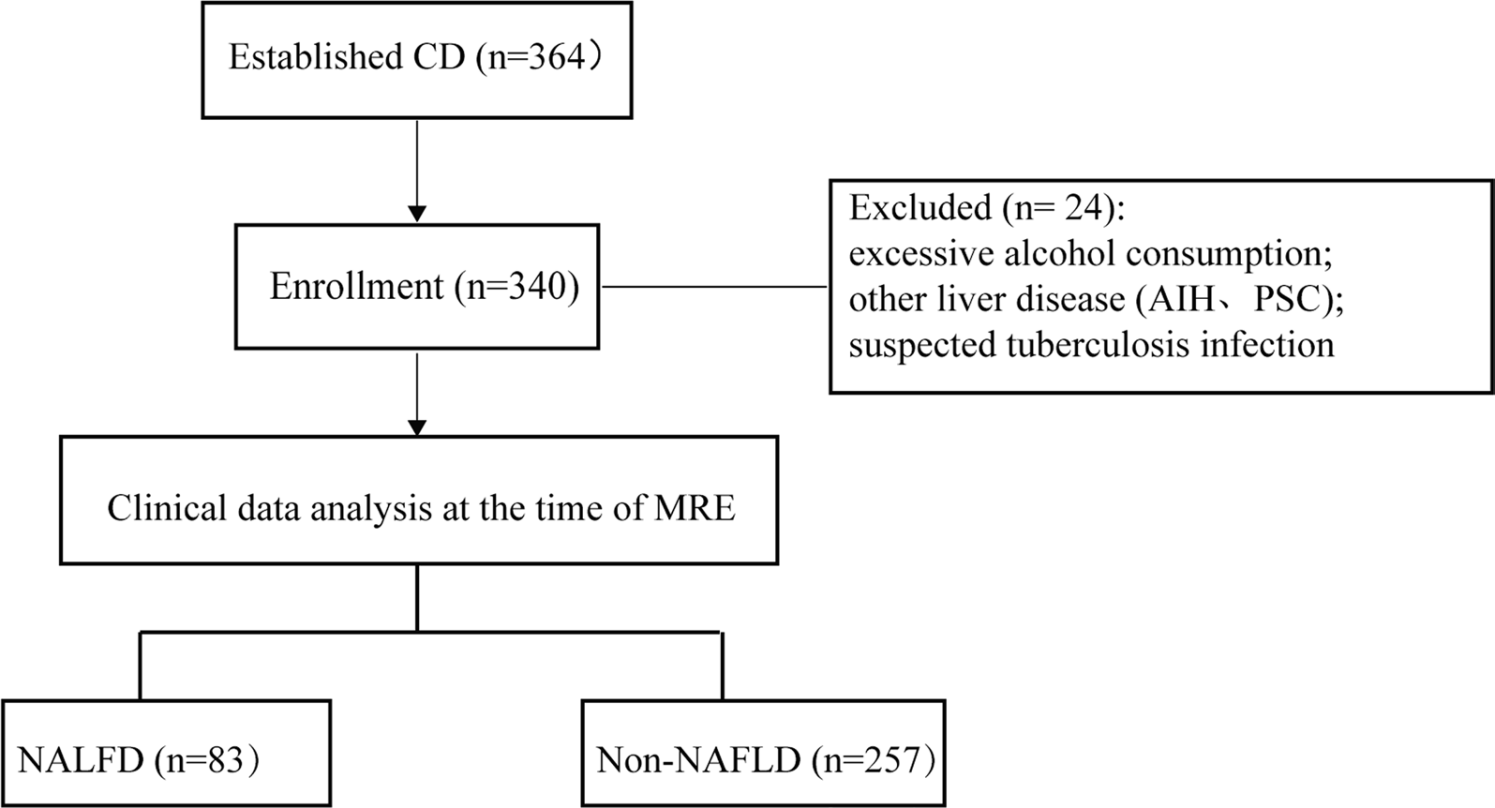
Flow diagram of the study design

After application of inclusion and exclusion criteria, there were 340 CD subjects who were eligible for analysis, 83 patients of whom were diagnosed with NAFLD according to the MR result. The prevalence of NAFLD among the CD patients of this Chinese cohort was 24.8% (83/340). A flow diagram illustrating the process is presented in Figure 1.

As shown in Table 1, the duration of CD was significantly longer than those without NAFLD (P = 0.01), though the gender and age at diagnosis were comparable between groups. The gender ratio was also nearly identical among groups. With regard to  BMI, those with NAFLD had a higher BMI than those without NAFLD (P < 0.01). Furthermore, there was no difference in the presence of metabolic syndrome and disease behavior or location.

**Table 1:** Demographics and clinical characteristics at baseline

	Non-NAFLD	NAFLD	P-value
	(n = 257)	(n = 83)	
Gender (M, %)	178(69.3)	64(77.1)	0.21
Age, year	30 ± 11	32 ± 11	0.07
BMI, kg/m^2^	19.6 ± 2.7	22.3 ± 4.5	** < 0.01**
Current smoker	9(3.5)	2(2.4)	1
MS	18(7)	29(34.9)	** < 0.01**
Disease duration, year	2(0.8–5)	4(1–7)	**0.01**
CDAI	134(73–251)	130(65–246)	0.57
Active CD	125(48.6)	36(43.4)	0.45
Disease location			
L1	98(38.1)	38(45.8)	0.25
L2	11(4.3)	4(4.8)	0.77
L3	148(57.6)	41(49.4)	0.37
P	161(62.6)	49(59)	0.6
Disease Behavior			
B1	150(58.4)	42(50.6)	-
B2	61(23.7)	25(30.1)	-
B3	46(17.9)	16(19.3)	0.4
CRP	0.6(0.5–5.6)	0.6(0.5–5.3)	0.72
Alb	46.7(41.8–50.2)	46.1(42.9–49.3)	0.98
PAB	220(166.5–263)	256(197.1–292.9)	** < 0.01**
ALT	14(10–22)	23.8(16–36)	** < 0.01**
AST	18(15–22)	22(17–27)	** < 0.01**
GGT	16(12.4–23)	26(15–41)	** < 0.01**
5-ASA	146(56.8)	49(59)	0.63
AZA	107(41.7)	41(49.4)	0.06
Corticosteroids	96(37.4)	38(45.8)	**0.04**
Prolonged anti-TNF-α antibody use	151(58.8)	51(61.4)	0.7
TNF-α (ELISA)	272.7 ± 320.8	222.9 ± 303.1	0.59
IL-6(ELISA)	5.3 ± 4.3	4.3 ± 2.8	0.39

Values presented as Mean ± SD with t-test; Median [P25, P75] with Wilcoxon rank sum test, or N (%) with Pearson's chi-square test

SD, standard deviation; BMI, body mass index; MS, metabolic syndrome; NAFLD, non-alcoholic liver disease; CRP, C- reactive protein; TP, total protein; Alb, albumin; PAB, pre-albumin; ALT, Alanine aminotransferase; AST, Aspartate aminotransferase; GGT, γ-glutamyl transpeptidase; 5-ASA, 5-Aminosalicylic acid; AZA, Azathioprine; TNF-α, tumor necrosis factor α; IL-6, Interleukin-6

### Laboratory result

As for laboratory examination, no significant differences were found in the C-reaction protein level (P = 0.72), or albumin level (P = 0.98). However, it is significantly different in the level of liver function enzyme, such as Alanine aminotransferase (ALT), Aspartate aminotransferase (AST), γ-glutamyl transpeptidase (GGT) (P < 0.01), as well as the level of pre-albumin (PAB) (P < 0.01).

### Medication

No differences were observed between the groups with regard to receiving prolonged anti-TNF-α antibody (P = 0.7), with 51 of 83 (61.4%) in the NAFLD cohort and 151 of 257 (58.8%) in the CD only cohort. Furthermore, there was no difference in use of 5-aminosalicylic acid (P = 0.63) and azathioprine (P = 0.06) in the 2 groups. However, the NAFLD group has significantly more patients who received corticosteroid treatment (p = 0.04).

### Model development

The results from multivariate logistic regression analysis showed that BMI (OR, 2.387, 95% CI 1.694–3.362; p < 0.0001), ALT (OR, 1.653, 95% CI 1.133–2.411; *P* = 0.0091), AST (OR, 0.629, 95% CI 0.423–0.938; *P* = 0.023), GGT (OR, 1.266, 95% CI 1.049–1.528; *P* = 0.0139), PAB (OR, 1.566, 95% CI 1.132–2.167; *P* = 0.0067) were robust predictors of risk of NAFLD, based on which, the final prediction model were developed. The model consisted of disease duration, BMI, ALT, AST, GGT and PAB and the diagnostic equation was: logit (P) = (− 6.865) + duration × (0.057) + BMI × (0.21) + ALT × (0.036) + AST × (-0.051) + GGT × (0.015) + PAB × (0.005) (Table 2).

**Table 2:** Multivariate analysis for factors associated with NAFLD in CD patients

Variable	Coefficient	OR (95%CI)	P-value
Duration BMI	0.0572 0.2098	1.059 (0.99–1.133) 2.387 (1.694–3.362)	0.0971 < 0.0001
ALT AST	0.0359 − 0.0514	1.653 (1.133–2.411) 0.629 (0.423–0.938)	0.0091 0.0230
GGT PAB	0.0155 0.0046	1.266 (1.049–1.528) 1.566 (1.132–2.167)	0.0139 0.0067
Intercept	− 6.8648	–	–

Values of OR > 1 indicate a direct relationship, values < 1 an inverse association

NAFLD, non-alcoholic fatty liver disease; CD, Crohn’s disease; OR, odd ratio; BMI, body mass index; ALT, Alanine aminotransferase; GGT, γ-glutamyl transpeptidase; PAB, Pre-albumin

**Fig. 2:**
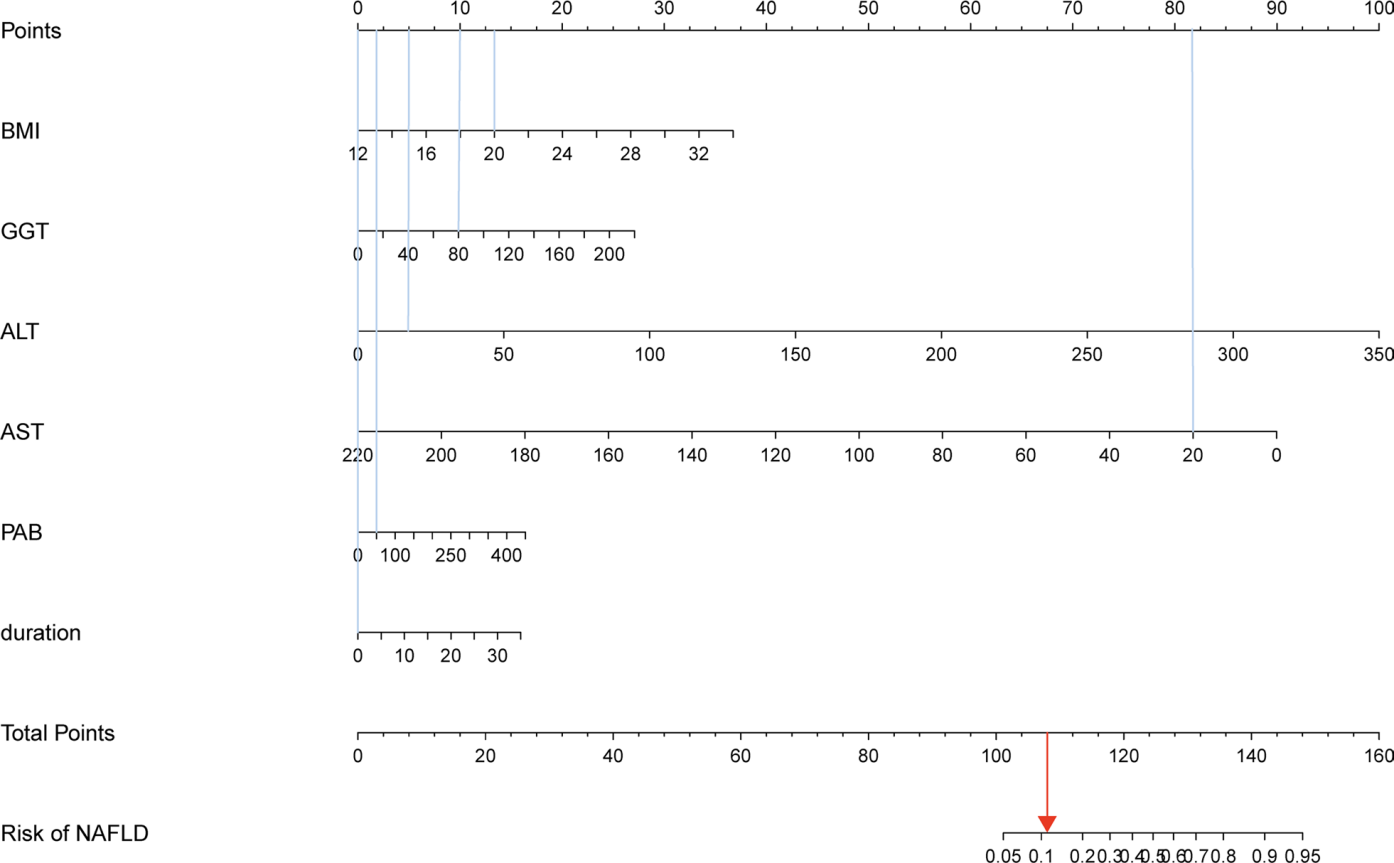
Nomograms for predicting risk of NAFLD. The usage of the nomogram is illustrated in a hypothetical patient with CD, a BMI of 20, GGT of 80 U/L, ALT of 30 IU/L, AST of 20 U/L, PAB of 50 mg/L and duration of 3 months upon admission (vertical blue lines). According to the nomogram, points for each parameter were 13, 10, 5, 82, 2 and 0 respectively. The total points added up to 112, which represented approximately 0.12 probability (vertical red line). NAFLD, non-alcoholic fatty liver disease; BMI, body mass index; ALT, Alanine aminotransferase; GGT, γ-glutamyl transpeptidase; PAB, Pre-albumin.

**Fig. 3:**
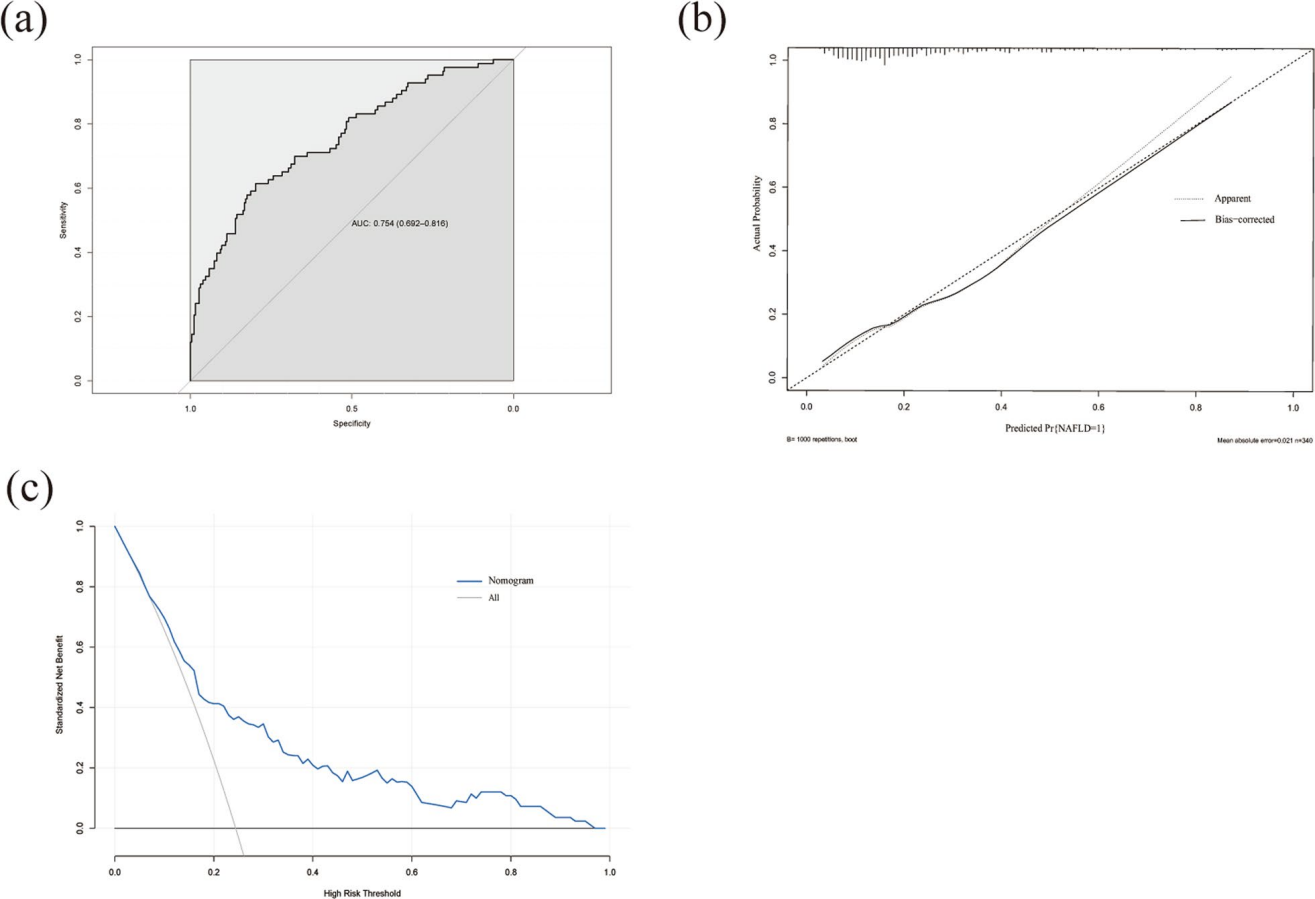
**a** The ROC curve and AUC of the nomogram; **b** Calibration plot of the nomogram; (3) Decision Curve (DCA)

A nomogram including these six parameters was presented to calculate the risk probability of NAFLD (Fig. 2). Receiver operating curve analysis shows that the area under ROC curve of model was 0.754 (Fig. 3a), indicating the diagnostic performance was good. The calibration curve showed that the mean error was 0.021 (Fig. 3b). DCA showed that within a very broad range for thresholds, the nomogram would bring net benefit in clinical practice (Fig. 3c).

## Discussion

Crohn’s disease is a remitting and chronic disease that often involves the small intestine [23]. Thus, a complication of malnutrition and malabsorption is frequently occurred. However, since a study first reported an association between “an expanded fatty liver” and “ulceration of the colon” in 1873 [24], there has been several studies conducted to further study the link between the diseases. The prevalence of NAFLD in CD is reported to vary between 8.2 to 39.5% [5, 5, 5], depending on the diagnostic tools and criteria applied. And previous studies have proposed several factors associated with the NAFLD, including ethnic, BMI, disease duration [15], anti-TNF-α therapy [26] and etc. However, these studies were mostly conducted in a western population and only discuss the association.

Our study collected a group of Chinese patients diagnosed with CD, who completed MR during hospitalization, based on which the liver fat was imaging and calculated. And several implications can be highlighted from this research. Firstly, the prevalence of non-alcoholic liver disease is estimated to be 24.8% in CD patients, according to the standard of MR-PDFF > 5.5%, higher than the overall prevalence of 17% in China and Japan area [27]. This is consistent with a previous study reported in Japan, which reported a prevalence of 21.8% [25]. Although the age between groups did not differ significantly, there was a trend that the patients with NAFLD were older than the other group. In our study, CD patients with NAFLD presented longer disease duration, which is in accordance with the previous study [6]. Longer disease duration may lead to multiple risk factors for NAFLD, such as chronic inflammation, dysbiosis of gut microbiota and hepatotoxic drugs. Specifically, the alternation of gut microbiota may play a pivotal role in the mechanistic link of IBD and NAFLD. Even though the etiology of inflammatory bowel disease remain partially understood, intestinal microbiota and their metabolites emerged as key actors in the pathogenesis of IBD [28, 29]. Likewise, NAFLD is associated with increased intestine permeability which can amplify many gut-derived effects, ending up with overgrowth of bacteria in these patients [30]. Besides, the NAFLD group had a higher BMI and more frequent presence of metabolic syndrome.

Another finding is that there is no significant difference in the long-term use of anti-TNF-α between groups. Ambiguous results have been reported in the previous research [11, 11]. However, neither of them evaluated the long-term effect of the drugs. In this study, we specifically document the times of patients receiving anti-TNF-α therapy, and did not find significantly difference in the occurrence of NAFLD in patients with prolonged anti-TNF-α use.

Liver fat content can be accurately and noninvasively detected through imaging examinations, such as ultrasound, computed tomography or magnetic resonance imaging. Among these tests to diagnose NAFLD, ultrasound and computed tomography (CT) share close sensitivity and reliably identify steatosis when the liver comprises ≥ 20% of fat. Although MRI can identify as little as 5% steatosis, the cost is much more higher [30]. Therefore, a clinical classifier is needed to evaluate the risk for NAFLD in a convenient, cost-effective manner, then to elevate patient compliance.

Applying logistic regression analysis, our model incorporates six parameters: BMI, PAB, ALT, AST, GGT and duration. The model has a high AUC value, which is indicative of greater sensitivity and specificity. The Hosmer–Lemeshow test shows that the model’s predicted value does not differ significantly from the actual one. The C-index value is 0.753, indicating good consistency of our model. The model’s calibration slope demonstrates a value of 0.834 after bias correction. The nomogram is a method with superiority widely used by oncologists to predict tumor prognosis. In current research, we have successfully created a visualized nomogram to predict the risk of developing NAFLD.

The current strategy of NAFLD therapy in general focus on dietary and lifestyle modification, with the aim to reduce weight, which may be not completely suitable for the IBD population due to the presence of malnutrition and uncontrolled inflammation. Although no clear guidelines have been established, the management of these patients should be individualized and guided by existing protocols for those without IBD.

The major advantages of our study include the diagnosis of NAFLD based on MR-PDFF, use of a large cohort of CD patients, first to evaluate the long-term effect of use of anti-TNF-α on NAFLD. Nevertheless, several limitations need to be addressed. First, due to the retrospective nature, we defined NAFLD through chart review rather than prospectively collected the liver content data. Second, this is a monocentric cross-sectional research, which may limit the application of the diagnostic model, yet lacking a control cohort to assess the predictive factors associated with progression of NAFLD. Third, we did not take the liver fibrosis into account, which is a robust predictor for disease-specific mortality in patients with histologically conformed NAFLD. Further studies are warranted to assess if and how the immune dysfunction plays a role in liver steatosis.

## Conclusion

In conclusion, our data report a comprehensive investigation of prevalence of NAFLD in Chinese population, which is high in chronic CD patients. And long-term use of anti TNF-α seems to have no effect on the progression of NAFLD in Chinese population. We also develop a nomogram to instruct clinical practice.

## Supplementary Information

**Additional file 1.** Supplementary data associated with this article.

## Data Availability

The datasets used and/or analyzed during the current study are included in its supplementary information files.

## Abbreviations

CD: Crohn’s disease
NAFLD: Non-alcoholic liver disease
IBD: Inflammatory bowel disease
MRE: Magnetic resonance enterography
T2DM: Type 2 diabetes mellitus
CKD: Chronic kidney diseases
BMI: Body mass index
AIC: Akaike’s information criterion
ROC: Receiver operating characteristic
DCA: Decision curve analysis
