# Correction to: Prevalence and predictors of non-alcoholic liver disease on MRI among patients with Crohn‘s disease

**DOI:** 10.1186/s12876-022-02297-8

**Published:** 2022-05-13

**Authors:** Qijin Hong, Jun Shen, Qi Feng, Qing Zheng, Yuqi Qiao

**Affiliations:** 1 Division of Gastroenterology and Hepatology, Shanghai Institute of Digestive Disease, NHC Key Laboratory of Digestive Diseases, Inflammatory Bowel Disease Center, Renji Hospital, School of Medicine, Shanghai Jiao Tong University, 160 Pu Jian Road, Shanghai, 200127 China; 2 Department of Radiology, Renji Hospital, School of Medicine, Shanghai Jiao Tong University, 160 Pu Jian Road, Shanghai, 200127 China

## Correction to: BMC Gastroenterology (2022) 22:183 https://doi.org/10.1186/s12876-022-02238-5

After publication of this article [1], the authors reported that also Qi Feng and Qing Zheng should have been denoted as corresponding author.

The original article [1] has been updated.
